# Survival Analysis of Patients With COVID-19 in India by Demographic Factors: Quantitative Study

**DOI:** 10.2196/23251

**Published:** 2021-05-06

**Authors:** Sampurna Kundu, Kirti Chauhan, Debarghya Mandal

**Affiliations:** 1 Department of Mathematical Demography and Statistics International Institute for Population Sciences Mumbai India

**Keywords:** survival analysis, COVID-19, patient data, Kaplan-Meier, hazard model, modeling, survival, mortality, demographic, India, transmission

## Abstract

**Background:**

Studies of the transmission dynamics of COVID-19 have depicted the rate, patterns, and predictions of cases of this pandemic disease. To combat transmission of the disease in India, the government declared a lockdown on March 25, 2020. Even after this strict lockdown was enacted nationwide, the number of COVID-19 cases increased and surpassed 450,000. A positive point to note is that the number of recovered cases began to slowly exceed that of active cases. The survival of patients, taking death as the event that varies by age group and sex, is noteworthy.

**Objective:**

The aim of this study was to conduct a survival analysis to establish the variability in survivorship of patients with COVID-19 in India by age group and sex at different levels, that is, the national, state, and district levels.

**Methods:**

The study period was taken from the date of the first reported case of COVID-19 in India, which was January 30, 2020, up to June 30, 2020. Due to the amount of underreported data and removal of missing columns, a total sample of 26,815 patients was considered. Kaplan-Meier survival estimation, the Cox proportional hazard model, and the multilevel survival model were used to perform the survival analysis.

**Results:**

The Kaplan-Meier survival function showed that the probability of survival of patients with COVID-19 declined during the study period of 5 months, which was supplemented by the log rank test (*P*<.001) and Wilcoxon test (*P*<.001) to compare the survival functions. Significant variability was observed in the age groups, as evident from all the survival estimates; with increasing age, the risk of dying of COVID-19 increased. The Cox proportional hazard model reiterated that male patients with COVID-19 had a 1.14 times higher risk of dying than female patients (hazard ratio 1.14; SE 0.11; 95% CI 0.93-1.38). Western and Central India showed decreasing survival rates in the framed time period, while Eastern, North Eastern, and Southern India showed slightly better results in terms of survival.

**Conclusions:**

This study depicts a grave scenario of decreasing survival rates in various regions of India and shows variability in these rates by age and sex. In essence, we can safely conclude that the critical appraisal of the survival rate and thorough analysis of patient data in this study equipped us to identify risk groups and perform comparative studies of various segments in India.

**International Registered Report Identifier (IRRID):**

RR2-10.1101/2020.08.01.20162115

## Introduction

The entire world has been greatly challenged by the sudden outbreak of COVID-19, as the human race has no remedial measures to combat the lethal impact of the disease. According to the World Health Organization (WHO), the global pandemic of COVID-19 is derived from SARS-CoV-2, a member of a large family of viruses, named coronaviruses; these viruses cause respiratory infections ranging from the common cold to high fever, leading to disease. This blue planet has witnessed many epidemics, such as that caused by severe acute respiratory syndrome coronavirus (SARS-CoV) from 2002 to 2003 and H1N1 influenza in 2009 [1], due to various pernicious viruses in the last two decades; however, COVID-19 is incomparable with previous epidemics because of the indomitable growth rate of the disease and its high fatality rate. China was the first country to experience high numbers of cases at the beginning of the pandemic; presently, Chinese authorities have “flattened the curve” with continuous testing and aggressive quarantine measures [2]. Outside China, South Korea was the country that had the largest initial outbreak; they managed to slow the spread of COVID-19 and flatten the curve without imposing lockdown in their country [3]. The only method used to slow and contain the outbreak in Korea was mass diagnostic testing and quarantining. The WHO declared that incorporating self-isolation, sanitizing, washing hands repeatedly and abstaining from touching the mouth, face, or nose to stop the spread of COVID-19 [4]. To combat transmission of the disease in India, the government declared a lockdown on March 25, 2020. However, the disease has spread rapidly across the entire country, and as of June 30, 202, there were 1,385,494 cases, with 32,096 deaths and 886,235 recoveries [5]. For a developing country such as India, the COVID-19 pandemic is a serious problem facing the nation, and the main sufferers are marginalized sections of society. Even after a strict lockdown was established nationwide, the number of cases increased and surpassed 450,000. However, the fatality rates later decreased, and several studies have shown that the lockdown did slow the rate of increase in a number of cases [6]. A positive point to be noted is that the number of recovered cases is slowly exceeding that of active cases.

In the study of dynamics of infectious diseases, compartmental models and the basic reproductive number (r_0_) have been observed to be the mostly commonly used over the past year [7]. Basic mathematical models such as the Gompertz, exponential, and logistic growth models have shown to be quite effective in understanding the growth patterns of the disease [8]. One of the main demerits of the Indian database for COVID-19 is the underreporting of cases due to misreporting and the lower number of tests [9]. Amid the growing number of deaths due to COVID-19, researchers worldwide have associated these deaths with additional important cofactors, namely the effect on the older population and the impact of pollution and smoking as well as the development of acute respiratory distress syndrome [10,11]. In one study [12], a district-level analysis showed that 92 districts in India are in red zones of the disease. These red zones are mostly found in the states of Maharashtra and Gujarat; in another study [13], it was predicted by the autoregressive integrated moving average (ARIMA) model that the number of cases will increase alarmingly.

Studies have described the impact of lockdown, the transmission dynamics of the disease, and forecasts of the pandemic. The survival of patients, taking death as the event that varies by age group and sex, is noteworthy. The aim of this study was to conduct a survival analysis to establish the variability in survivorship by age group and sex at different levels, that is, the national, state, district, and patient levels. This quantitative analysis (with analysis of data from patients with COVID-19) is exceptional not only for its gravity and pertinence but also for its subtle nuances and penetrating approach.

## Methods

### Data and Analysis

**Figure 1:**
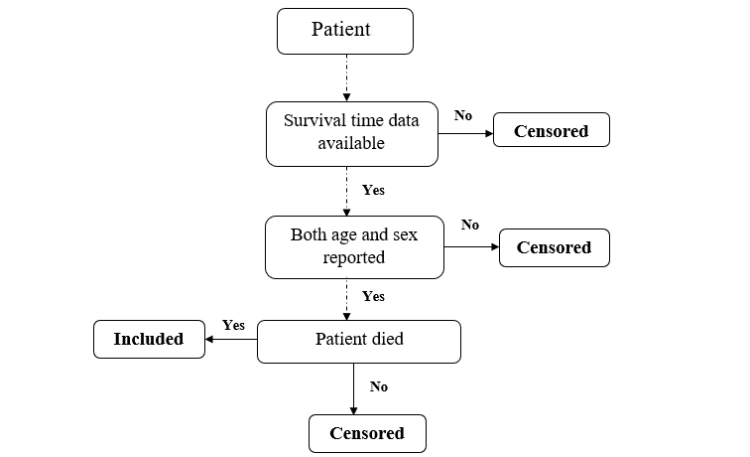
Flowchart of the inclusion of patients in the study.

The data for this study were retrieved from the data sharing portal of India [5]. Patient-level data, consisting of time-to-event data, were used for the study. Here, the study period is from the date of the first case report in India, which was January 30, 2020, to June 30 of that year (ie, 5 months or 150 days). The entry point of each patient was different, and the event of interest in this study was death. If this event had not occurred, the survival time was taken to be censored. Due to the amount of underreported data and dropping of missing columns, a total of 26,815 sample patients were considered. The inclusion criteria for each patient were the date on which they tested positive for COVID-19, the date of the change of status, and reported age and sex. Survival time was computed by taking the difference between the date on which each sample patient tested positive for the infection and the date of the change of status. A flowchart of the selection of patient data for the study is shown in Figure 1.

The Kaplan-Meier survival estimator method, Cox proportional hazard model, and multilevel survival model were used to perform the survival analysis. Firstly, the Kaplan-Meier survival estimator method was used to estimate the survival function from the survival data. To compare the survival functions for different groups (ie, by sex, age group, and region), the log-rank test and Gehan-Breslow-Wilcoxon test were used. To estimate the survival functions in the presence of various covariates, the Cox proportional hazard model was used, with sex, age, and region as the covariates, assuming that the hazard is independent of time. The Cox proportional hazard model can be expressed as below:

h(t) = h_0_(t)exp(*β*_1_sex + *β*_2_age + *β*_3_region)

where *t* represents the survival time and *h*(*t*) is the hazard function determined by a set of 3 covariates (sex, age, region). The coefficients (*β_1_, β_2_, β_3_*) measure the impact (ie, the effect size) of the covariates. The term *h*_0_* *is called the baseline hazard, and it corresponds to the value of the hazard if all values of x*i* are equal to zero.

Finally, multilevel mixed effects survival analysis was performed, as clustering of lower level units at higher level units is a common scenario in such studies. Here, patients were clustered at the district level; then, the districts were clustered at the state level, and all the states were clustered at the national level. We considered *i*-1,2,…..,*N* clusters (eg, states and districts), with each cluster having *j = 1,2,…,n_i_* patients. Let *S_ij_* be the true survival time of the *j^th^* patient in the *i^th^* cluster, *T_ij_* = min(*S_ij_*,*C_ij_*) be the observed survival time, and *C_ij_* be the censoring time. The proportional-hazards mixed-effects survival model can be written as below:



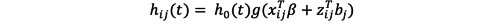



where *h*_0_(*t*) is the baseline hazard function of a standard parametric model (eg, here, we use Weibull at each level because according to the Akaike information criterion, it is the most appropriate model to use). Therefore, a 3-level cluster analysis will help eliminate the variability at each level due to intercorrelation between the units and can provide better estimates of the survival function.

### Ethical Approval

The research was conducted using a publicly available database. The authors assert that all procedures contributing to this work comply with the ethical standards of the relevant national and institutional committees in human experimentation and with the Helsinki Declaration of 1978 as revised in 2008.

## Results

**Figure 2:**
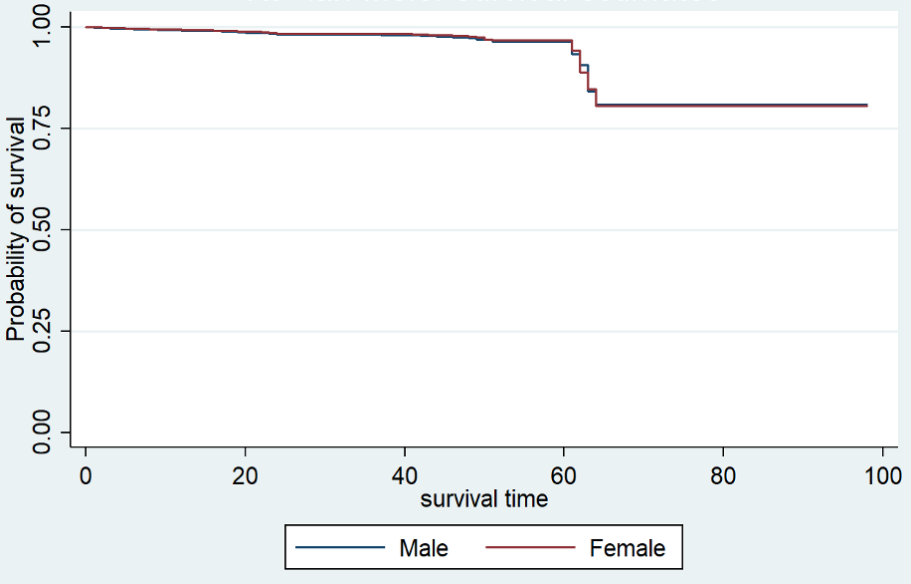
Kaplan-Meier estimate of survival of patients with COVID-19 in India by gender.

Kaplan-Meier estimates were obtained initially to estimate the survival functions of patients in India with COVID-19 by sex, age group, and region. It can be seen from Figure 2 that the survival curves from the Kaplan-Meier estimator for male and female sex are almost the same. In Table 1, it can be observed that according to both the log rank test (*P*>.001) and Wilcoxon test (*P*>.001) for comparing the survival functions, the difference is not significant, indicating that there is no significant difference between the survival curves of male and female patients with COVID-19.

**Table 1:** Kaplan-Meier estimator: comparison of survival functions for patients with COVID-19 in India by sex.

Test	*χ^2^ (1)*	*P* value
Log rank	1.6	.20
Wilcoxon	2.6	.11

**Figure 3:**
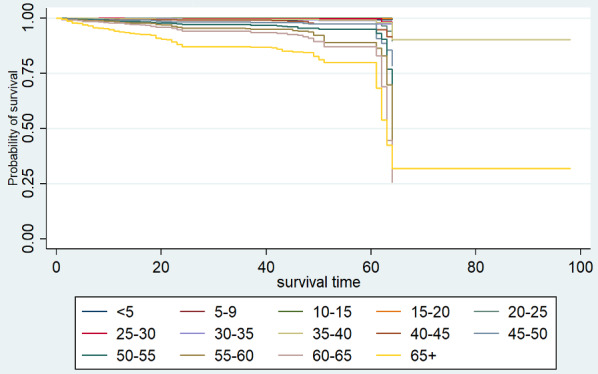
Kaplan-Meier estimate of survival of patients with COVID-19 in India by age group (years).

As shown in Figure 3, the survival curves from the Kaplan-Meier estimator by 5-year age group are significantly different. This result was further supplemented by the log rank test (*P*<.001) and Wilcoxon test (*P*<.001) for comparing the survival functions (Table 2); both tests gave highly significant results, indicating that there are significant differences among the survival curves of various age groups.

**Table 2:** Kaplan-Meier estimator: comparison of survival functions for patients with COVID-19 in India by age group.

Test	*χ^2^ (13)*	*P* value
Log rank	1302.7	<.001
Wilcoxon	1072.26	<.001

**Figure 4:**
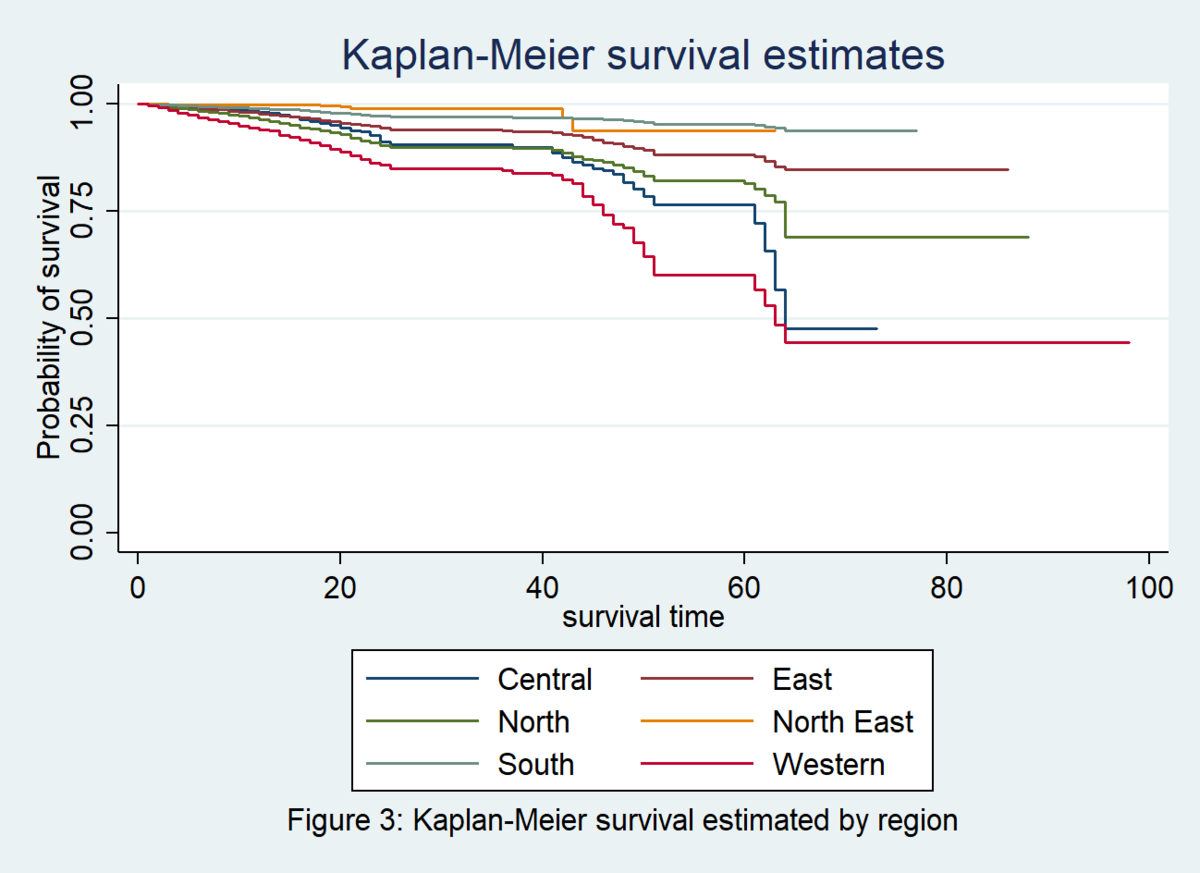
Kaplan-Meier estimate of survival of patients with COVID-19 in India by region.

**Figure 5:**
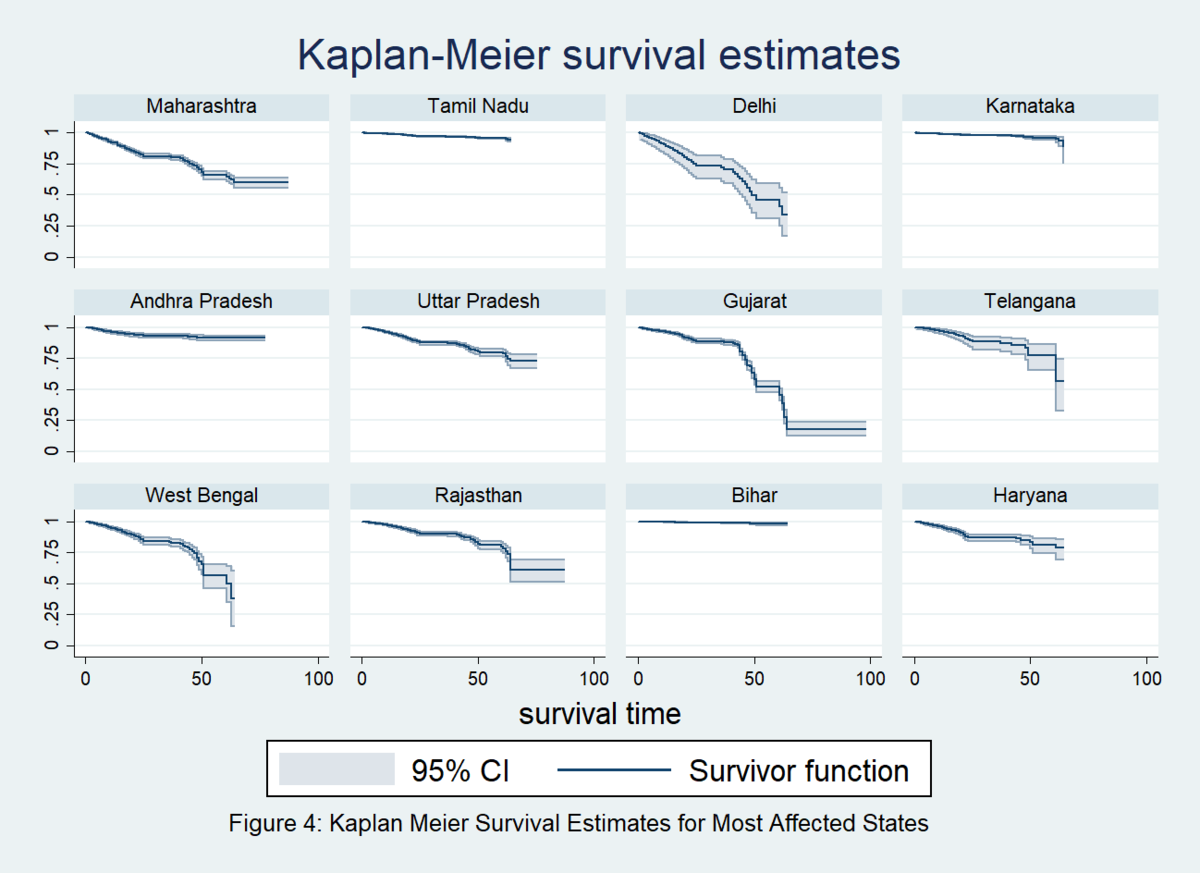
Kaplan-Meier survival estimates for the states in India most affected by COVID-19.

Figure 4 depicts that the survival curves from the Kaplan-Meier estimator for different regions of India are significantly different. From Table 3, it can be inferred that the log rank test (*P*<.001) and Wilcoxon test (*P*<.001) for comparing the survival functions are both highly significant, indicating that there are significant differences among the survival curves by region due to regional variations. Therefore, we can find that both age and region are significantly associated with the survival rate of COVID-19 without adjusting for other covariates. Figure 5 depicts a comparison of the survival curves among the states most affected by COVID-19, which shows the low survival rates in Maharashtra, Delhi, Gujarat, West Bengal, and Rajasthan.

**Table 3:** Kaplan-Meier estimator: comparison of survival functions for patients with COVID-19 in India by region.

Test	*χ^2^ (5)*	*P* value
Log rank	1997.43	<.001
Wilcoxon	1175.27	<.001

Table 4 presents the results of the survival analysis using the Cox proportional hazard model and reiterates that male patients with COVID-19 have a 1.14 times higher risk of dying than female patients (hazard ratio [HR] 1.14; SE 0.11; 95% CI 0.93-1.38). Coming to the agewise comparison, we observed that patients aged 45-49 years to ≥65 years had a 5.83, 10.08, 15.31, 22.03, and 39.21 times higher risk of dying due to COVID-19 than those in the age group of 0-5 years, respectively. The highest risk of death from the disease was among people in the ≥65 years age group, with 39.2 times higher risk (*P*<.001) but a larger confidence interval (95% CI 9.73-157.97). When analyzing the survival curves by region, it can be seen that patients in the East, North East, and South Indian regions were at 59%, 14%, and 26% lower risk of dying from COVID-19 infection than those in Central India, respectively, whereas patients in the West Indian region had a 1.9 times higher risk of dying than those in Central India.

A multilevel survival model was applied at the overall national level (Weibull regression) and the state and district level (mixed effects Weibull regression), taking age group and sex as the covariates. The results of the multilevel survival analysis, that is, after controlling the variability due to clustering of the lower level at the higher level, can be seen in Table 5; the hazard ratio at the India level shows that male patients are at 1.27 times higher risk than female patients of dying from COVID-19 (HR 1.27; SE 0.13), which is almost the same at the state level (HR 1.32; SE 0.13) as well as the district level (HR 1.21; SE 0.13). Significant variability in survival was observed for the age groups older than 45 years. At all levels, we found that the hazard ratio increased with increasing age but decreased across each level. For instance, in the ≥65 years age group, the patient is at 39.3 times higher risk of dying overall than a patient in a younger age group (HR 39.3; SE 27.94); meanwhile, at the state level, the hazard ratio is 32.28, and that at the district level is 23.55. Now, from the variance of the errors of model, we can infer that the heterogeneity is greater at the district level (*σ_e_^2^* 6.85; SE 1.35) than at the state level (*σ_e_^2^* 2.28; SE 0.83).

**Table 4:** Cox proportional hazard model showing unadjusted hazard ratios and 95% confidence intervals for deaths occurring due to COVID-19 in India.

Independent variable			Unadjusted hazard ratio (SE)		95% CI
**Sex**					
	Female	Reference		N/A^a^	
	Male	1.14 (0.11)		0.93-1.38	
**Age group (years)**					
	0-4	Reference		N/A	
	5-9	0.00 (N/A)		N/A	
	10-14	0.00 (N/A)		N/A	
	15-19	0.74 (0.64)		0.14-4.03	
	20-24	0.85 (0.67)		0.18-4.01	
	25-29	0.87 (0.68)		0.19-4.01	
	30-34	1.44 (1.09)		0.32-6.36	
	35-39	1.52 (1.15)		0.34-6.73	
	40-44	3.66 (2.69)		0.87-15.44	
	45-49	5.83 (4.24)^b^		1.40-24.22	
	50-54	10.08 (7.27)^c^		2.45-41.42	
	55-59	15.31 (11.00)^c^		3.74-62.59	
	60-64	22.03 (15.81)^c^		5.40-89.90	
	≥65	39.21 (27.88)^c^		9.73-157.97	
**Region**					
	Central				
	East	0.59 (0.05)^c^		0.50-0.70	
	North	0.98 (0.09)		0.82-1.16	
	North East	0.14 (0.04)^c^		0.08-0.24	
	South	0.26 (0.02)^c^		0.22-0.31	
	West	1.90 (0.16)^c^		1.61-2.23	

^a^N/A: not applicable.

^b^Significant at a 1% level of significance.

^c^Significant at a 0.1% level of significance.

**Table 5:** Multilevel survival analysis by hazard ratio at the national, state, and district levels for deaths among patients with COVID-19 in India.

Independent variable			Level, hazard ratio (SE)				
			National		State^a^		District^b^
**Sex**							
	Female	Reference		Reference		Reference	
	Male	1.27 (0.13)^c^		1.32 (0.13)^c^		1.21 (0.13)	
**Age group (years)**							
	0-4	Reference		Reference		Reference	
	5-9 years	0.00 (0.00)		0.00 (0.00)		0.00 (0.00)	
	10-14	0.00 (0.00)		0.00 (0.00)		0.00 (0.00)	
	15-19	0.71 (0.62)		0.86 (0.75)		0.49 (0.45)	
	20-24	0.80 (0.63)		0.84 (0.66)		0.68 (0.54)	
	25-29	0.81 (0.63)		0.91 (0.71)		0.71 (0.56)	
	30-34	1.26 (0.96)		1.53 (1.16)		1.22 (0.93)	
	35-39	1.22 (0.94)		1.37 (1.05)		1.13 (0.87)	
	40-44	3.36 (2.47)		4.00 (2.94)		3.37 (2.49)	
	45-49	5.20 (3.78)^c^		6.22 (4.52)^c^		4.89 (3.56)^c^	
	50-54	8.53 (6.16)^d^		10.70 (7.73)^d^		7.77 (5.63)^c^	
	55-59	13.99 (10.06)^d^		14.76 (10.62)^d^		11.49 (8.29)^d^	
	60-64	20.52 (14.74)^c^		18.40 (13.23)^c^		14.19 (10.24)^c^	
	≥65	39.30 (27.94)^c^		32.28 (23.00)^c^		23.55 (16.84)^c^	

^a^Error variance: 2.28 (0.83).

^b^Error variance: 6.85 (1.35).

^c^Significant at a 1% level of significance.

^d^Significant at a 0.1% level of significance.

## Discussion

Epitomizing the whole study, it is notable that in the stipulated time period, our observation clearly revealed that the survival rate was continually declining, and to date, that trend has not abated. It is worth mentioning that age, sex, and regional variability were important determinants at each step. Also, from this study, it is very clear that the male population in India is more vulnerable to COVID-19, likely due to prevalent comorbidities and the dominant presence of men outside the home (also, our data support the fact that the survival rate of the female population is higher). This study also traced a different pattern for India than for other countries, as the younger population is greater in our country than in most countries where the number of affected people is numerous.

In this study, we strived to identify reliable features associated with survival patterns, and we inevitably scanned the roles of sex, age, and regional variability as controlling factors of the survival rate. For the survival analysis, the study period was 5 months, with death being the event of interest in our analysis. As we evaluated the Kaplan-Meier survival function, we observed that the probability of survival continually declined during the study period of 5 months. During the study period, no stabilization could be observed. Female patients were found to have better survival rates compared to their male counterparts, as is evident from the Cox proportional hazard results, which may be due to sex differentials in cellular compositions and the immunological microenvironment of the lungs [14,15]. Although we only observed a miniscule difference in the survival curves of male and female patients, it was stated in earlier studies that men with COVID-19 are at higher risk of death and health outcomes, independent of age [16], as men have greater disease burden (diabetes, hypertension, or cardiovascular diseases); therefore, men have shown markedly increased risk of developing severe COVID-19 in comparison to women. Also, a greater proportion of the confirmed cases are male rather than female; this finding is expected in a country with a gender hegemony in which work participation, mobility, and migration are predominately higher for men than for women [17], which makes men more vulnerable to the infection.

Significant variability was observed among age groups, as is evident from all the survival estimates, which show that with increasing age, the risk of dying from COVID-19 increases [18]. It was reported in a study that among comorbid patients with COVID-19, nearly 21% had hypertension, 11% had diabetes, and 7% had cardiovascular disease, which increased their risk of mortality [19]. In contrast to data from other countries, in India, only 15% of confirmed cases are aged >60 years, and the majority of these patients are in the age bracket of 25-59 years; this is most probably because the older population is the most affected by this pandemic and India has a fairly young population, which may contribute to a lower case fatality rate [9]. Approximately 84% of the patients with COVID-19 were men, and 82% patients overall were above 40 years of age, as reported in an Indian Council of Medical Research study [20]. India is one of the largest countries in the world, and it is highly diverse in every respect. Every province posseses its own demographic features, typical climatic character, and above all, its own lifestyle. Needless to say, these factors play a pivotal role. Therefore, variation in survival rate is easily traced. Although Western and Central India show continually decreasing survival rates in the framed time period, Eastern, Northeastern and Southern India show slightly better results in terms of survival. Maharashtra, Gujarat, Delhi, Rajasthan, and West Bengal showed alrmingly low survival rates as well.

Finally, this study has depicted a grave scenario of continual degradation of the COVID-19 survival rate in various regions. In essence, we can safely conclude that critical appraisal of the survival rate and thorough analysis of patient data in this study equipped us to identify risk groups and perform comparative studies of various segments of the population in India.

## Abbreviations

ARIMA: autoregressive integrated moving average
HR: hazard ratio
SARS-CoV: severe acute respiratory syndrome coronavirus
WHO: World Health Organization
